# Abdominal rhabdoid tumor presenting with symptomatic spinal epidural compression in a newborn. A case report

**DOI:** 10.3389/fped.2023.1337760

**Published:** 2024-01-12

**Authors:** Shana Montalto, Michela Di Filippo, Valeria Capra, Carla Manzitti, Angela Rita Sementa, Patrizia De Marco, Marzia Ognibene, Fiammetta Sertorio, Stefania Sorrentino

**Affiliations:** ^1^ Pediatric Infectious Diseases Unit, IRCCS IstitutoGianninaGaslini, Genoa, Italy; ^2^ Department of Neuroscience, Rehabilitation, Ophthalmology, Genetics, Maternal and Child Health, University of Genoa, Genoa, Italy; ^3^ Genomics and Clinical Genetics Unit, IRCCS Istituto Giannina Gaslini, Genoa, Italy; ^4^ Oncology Unit, IRCCS Istituto Giannina Gaslini, Genoa, Italy; ^5^ Department of Pathology, IRCCS IstitutoGianninaGaslini, Genoa, Italy; ^6^ Medical Genetics Unit, IRCCS Istituto Giannina Gaslini, Genoa, Italy; ^7^ Radiology Unit, IRCCS Istituto Giannina Gaslini, Genoa, Italy

**Keywords:** rhabdoid tumor, spinal compression, newborn, case report, oncological emergency

## Abstract

The occurrence of an abdominal tumor invading the spinal canal and causing symptoms of epidural compression is rare in an infant, and exceptional at birth. Peripheral neuroblastic tumors are by far the most common cause. Emergency chemotherapy is commonly curative, though permanent sequelae are possible. Although other malignancies may be involved, no case of rhabdoid tumors at birth has been reported. We describe the case of a neonate who presented symptoms of spinal epidural compression at birth secondary to a rhabdoid tumor. As expected with this highly malignant tumor, the patient experienced a rapidly progressive clinical course and died within three months of diagnosis.

## 1 Introduction

The occurrence of symptoms of spinal epidural compression (SEC) in an otherwise healthy child constitutes an emergency requiring prompt diagnosis and appropriate treatment. It is the result of an either benign or malignant paravertebral tumor entering the spinal canal through intervertebral foramina (1). Peripheral neuroblastic tumors (PNTs) are by far the commonest histotypes leading to SEC (2, 3). As PNTs are the most frequent tumor in infancy (2), the probability of their etiologic involvement when SEC occurs in the first year of life is even higher (4). Tumor-related SEC is rarely detected in a newborn, with approximately 15 cases described so far, almost all diagnosed with a PNT (5, 6). Here, we describe the case of a newborn presenting at birth with symptomatic SEC; the case was initially thought to be a PNT but was eventually diagnosed as a malignant rhabdoid tumor (MRT). This is possibly the first reported case of this association at birth.

## 2 Case description

A female Caucasic newborn was normally delivered by a 28-year-old woman after 38 (+5) weeks of an uncomplicated pregnancy. Prenatal ultrasound examinations did not show abnormal findings. Birth weight was 3,170 g. The mother had previously had two spontaneous pregnancies, interrupted for undefined reasons at the 5th and 28th (+4) weeks of gestation. The parents were unrelated and healthy. Family history included breast cancer in a paternal aunt, intestinal polypoid tumor in the paternal grandfather and lung cancer in the paternal grandmother, a heavy smoker*.* Physical examination at birth revealed a palpable right lumbar paraspinal, mild facial dysmorphism with sloping forehead and dysplastic ears, and two hernias of the abdominal wall (one laterally and one more anteriorly visible only during crying). In addition, neurological examination showed decreased limb strength and hypoactive reflexes of patellar tendons. No other symptoms were present at birth.

### 2.1 Diagnostic assessment, therapeutic intervention, follow-up and outcomes

**Figure 1:**
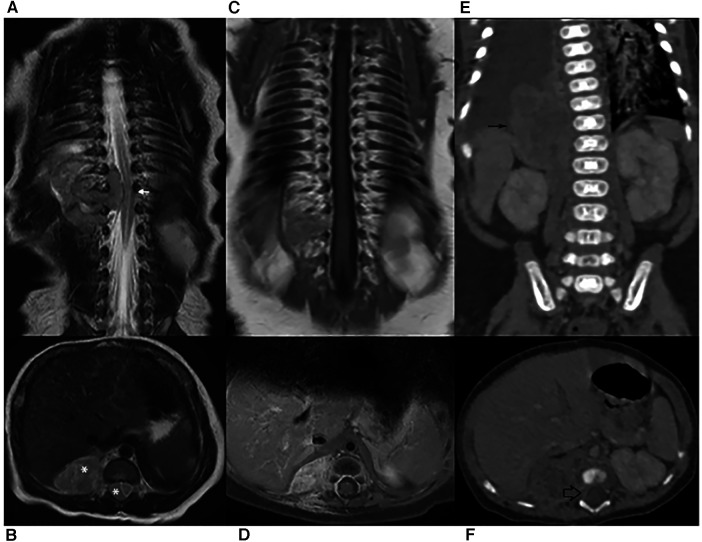
(**A**–**B**) MR scan on diagnosis. Coronal and transverse T2-weighted images showing spine dislocation and compression (white arrow). No significant spinal hyperintensity is seen, although the classical dumb-bell shape of the lesion (*) erases the epidural fat. (**C**–**D**) MRI after chemotherapy. Coronal and Transverse Gd-enhanced T1-weighted images: only mild enhancement is shown. The lesion had markedly decreased and the spinal cord had regained its normal position. (**E**–**F**) CT on relapse. Both abdominal (thin black arrow) and intra-canal (empty black arrow) portions are increased in volume.

Spinal magnetic resonance imaging (MRI) was performed on the second day of life and documented a 36 × 21 × 19 mm paravertebral lesion infiltrating the D10-L1 intervertebral foramina and invading the spinal canal, with displacement of the spinal cord (Figures 1A,B). Laboratory tests, including urinary catecholamine metabolite excretion, were normal except for a twice-normal level of lactate dehydrogenase. No secondary lesions were detected by thoracic computed tomographic (CT) scan and brain MRI. Both clinical and imaging features were strongly suggestive of a PNT. Following multidisciplinary discussion, the decision was taken to avoid a neurosurgical approach and to perform a tru-cut biopsy of the extraspinal tumor (day 4). The same day, chemotherapy consisting of the carboplatin-etoposide association was initiated on an emergency basis. Surprisingly, the histological report on the biopsy documented a tumor with rhabdoid morphology, polyphenotypical immunohistochemical profile and loss of *SMARCB1* (INI1) expression, strongly consistent with Malignant Extrarenal Rhabdoid Tumor (MERT).Microscopic examination revealed two fragments of 4 and 6 mm in length. The tissue was entirely neoplastic and featured large, polygonal cells with eccentric vesicular nuclei, prominent nucleoli and abundant eosinophilic cytoplasm, with consequent “rhabdoid” morphology. The neoplastic cells showed numerous mitoses and karyorrhexis figures. Immunohistochemical analysis was carried out by means of the available panel of antibodies. Mesenchymal, epithelial and neural antigens and other antibodies were tested, namely: Vimentin, HHF35, SMA (smooth muscle actin), desmin, Cytokeratins (CK AE1/AE3, CK7), EMA, CD31, CD34, pS100, ALK, CD20, CD43, CD117, CD56, MPO, NSE, Synaptophysin, NB84, TH, MAP-2, WT1, CD99, and BAF47. The neoplastic cells showed a polyphenotypic immunoreactivity pattern, expressing diffuse positivity for Vimentin, strong positivity for EMA, strong and diffuse positivity for SMA and HHF35, strong and diffuse CD99, focal pS100 and NSE and diffuse MAP-2 positivity. The cells showed loss of *SMARCB1* expression (INI1/BAF47 negativity). All the other markers tested were negative.The overall histological picture—as far as discernible on a small biopsy—was that of a tumor of the family of the *SMARCB1*-deficient tumors. Many tumors with polyphenotypic immunohistochemical expression and loss of *SMARCB1* expression were taken into consideration in the differential diagnosis, with particular reference to proximal-type epithelioid sarcoma, as this last entity shows overlapping features with Rhabdoid Tumor (7, 8). In our case, a few immunohistochemical features (such as negativity for CD34) together with the clinical setting (the extremely young age of the patient being a major consideration) were strongly in favor of the diagnosis of extrarenal RT. Consequently, the therapeutic protocol appropriate for this histology was adopted (*EpSSG NRSTS 2005*). Of course, on a small biopsy it was not possible to ascertain whether the tumor was exclusively composed of cells with rhabdoid morphology (a MERT- Malignant Extrarenal Rhabdoid Tumor) or featured focal areas within a parent neoplasm in which a clear line of differentiation could be documented (Composite Extrarenal Rhabdoid Tumor, or CERT) (7, 8). Our diagnosis was also supported by the patient's dismal outcome.

To exclude RT predisposition syndrome, which occurs in 15% of these patients, the INI1 gene Sanger sequencing of blood patient's DNA was performed. Constitutional DNA was also used for MLPA analysis to detect exonic gain/loss of the IN1 gene. However, both analyses showed no pathogenic alterations (4). In addition, given the dysmorphic features detected at birth, array CGH analysis was carried out on the patient's and parents' blood; this identified a 10q21.3 deletion of 81.28 kb *(68,415,590–68,496,866).* transmitted by the mother that resulted being a CNV.

**Figure 2:**
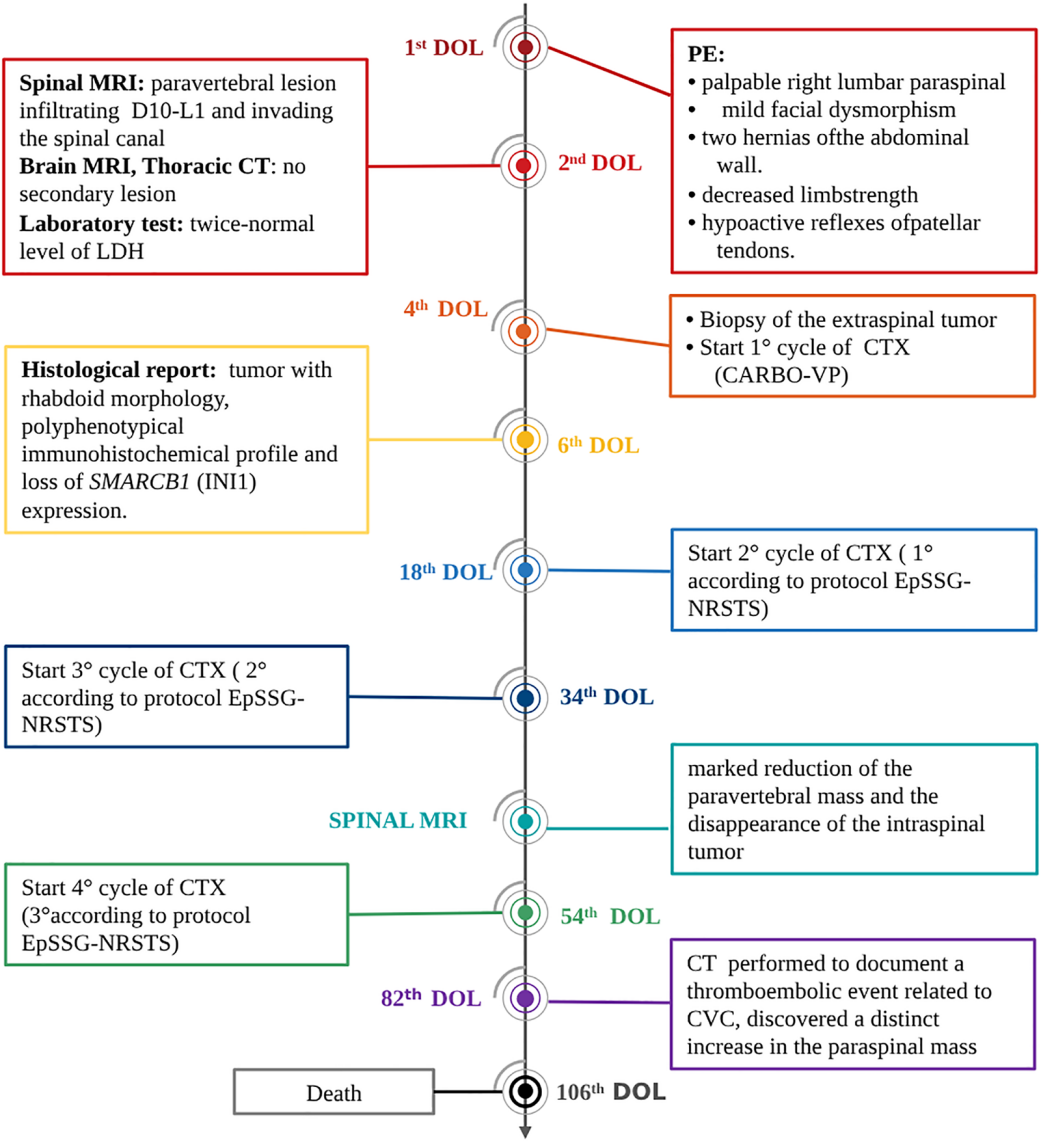
Timeline of interventions and outcomes. DOL, day of life; CTX, chemotherapy; PE, physical examination.

Two additional chemotherapy courses *(Cycle A: vincristine, cyclophosphamide, doxorubicin; Cycle B: cyclophosphamide, carboplatin, etoposide)* were carried out. At almost 2 monthsold, MRI revealed a marked reduction of the paravertebral mass and the disappearance of the intraspinal tumor (Figures 1C,D). One month later, and after another cycle of chemotherapy (*Cycle A)*, a CT scan performed to document a thromboembolic event related to the central venous catheter, discovered a distinct increase in the paraspinal mass (Figures 1E,F). This was soon followed by rapidly progressing respiratory distress secondary to massive pleural effusion, which proved positive for neoplastic cells. In the subsequent few days, the patient markedly deteriorated, dying on the 106th day of life (Figure 2).

## 3 Discussion and conclusion

Although a variety of pediatric tumors may occasionally present with SEC associated to a paravertebral mass, PNTs are by far the commonest causative hystotype (1, 2). Indeed, 5%–15% of children diagnosed with a PNT present with symptoms of SEC; more than half of these lesions are diagnosed in the first year of life, and, exceptionally, even in neonates (3–6). In the majority of neonates the first symptom was motor deficit, followed by bladder and/or bowel dysfunction. At the last follow-up, they almost invariably suffer the burden of permanent sequelae, in particular motor deficit, with only one patient that had no long-term sequelae (3–6).

As SEC may evolve into paraplegia, chemotherapy must be promptly administered to avoid the establishment of severe sequelae (1). There is worldwide consensus that treatment can be initiated before the histological diagnosis is made, provided that adequate tumor specimens have been secured for pathological and biological investigations. In our case, given the patient's age, the location of the lesion and the imaging characteristics, the tumor was deemed to be a probable PNT, despite the normal urinary catecholamine levels and the patient's dysmorphic features (unusual in PNTs). In the hypothesis of a PNT, the guidelines of the SIOPEN Group (9, 10) state that chemotherapy should be preferred to a neurosurgical approach. However, 48 h after the initiation of therapy, the histological report documented an RT. The chemotherapy program was therefore changed appropriately. Not unexpectedly, the tumor mass underwent rapid progression, resulting in death.

MRT is a highly aggressive neoplasm of infancy and childhood originally described in the kidney and Central Nervous System (CNS) (Atypical Teratoid Rhabdoid Tumors—ATRT). The clinico-pathological spectrum includes other organs, extrarenal soft tissues and skin. The name of these tumors derives from the rhabdoid appearance of the tumor cells, which have large, vesicular nuclei, prominent central eosinophilic nucleoli and abundant eosinophilic cytoplasm. MRT is a polyphenotypic neoplasm with coexpression of Vimentin, at least one epithelial marker (such as Cytokeratin—CK or EMA) neural or neuroectodermal markers such as pS100, GFAP, NSE and synaptophysin, and mesenchimal markers such as MSA, desmin, CD99 and SMA. Myogenin, myoglobin, human melanoma black 45 (HMB-45), chromogranin and CD34 are typically absent. About 75% of MRTs harbor deletions or mutations of the SMARCB1 gene at chromosome 22q11.2 (7, 8, 11). *SMARCB1* (SWI/SNF-related, matrix associated, actin-dependent regular of chromatin, subfamily B, member 1) is the protein product of the gene *SMARCB1* (also known asINI1 and SFN5) located on chromosome 22q11.2. INI1 is a core subunit of the SW1/SNF ATP-dependent chromatin remodeling complex and is ubiquitously expressed in the nuclei of normal cells; it is thought to function as a tumor suppressor (11). Germline *SMARCB1* mutations occur in both familial and sporadic cases. Immunohistochemical analyses for the *SMARCB1* protein in MRT of the CNS, kidney and extrarenal soft tissue have shown that absence of protein expression by immunohistochemistry(IHC) (INI1, BAF47) correlates with deletion and mutation of the *SMARCB1* gene. The absence of *SMARCB1* protein has been detected in a variety of soft tissue tumors, in CNS embryonal tumors without rhabdoid phenotype and in many other entities, indicating the existence of a wider family of *SMARCB1-*deficient tumors.

The differential diagnosis includes many tumors (Desmoplastic Small Round Cell Tumors—DSRCT, Rhabdomyosarcoma, Synovial sarcoma with rhabdoid features, rhabdomyomatous Wilms' Tumor (12) and, particularly, epithelioid sarcoma, because of the morphological and immunohistochemical overlap of these two tumors. The relationship between these two entities has not yet been clarified; although in the early literature there are contentions that these tumors fall within the same spectrum (13), strong evidence supports the interpretation that proximal-type epithelioid sarcoma and MERT are two distinct entities (14–17). The differential diagnosis is essentially based on clinic-pathological features (age, site), IHC (CD34 reactivity, analysis of specific markers of skeletal muscle differentiation etc.) and genetic features.

As usually occurs in the case of MRT, the neoplastic cells of our patient presented genetic alterations affecting the *INI1/hSNF5/SMARCB1* tumor-suppressor gene located 22q11.2. Although several treatment regimens have been tested to improve the grim outcome of MRT patients, less than 30% survive, with a median survival time of 10 months (18–20). Perinatal RTs have an even worse prognosis (20, 21). In the case of extracranial RT, young age (<1 year) and male gender are also negative prognostic factors (20).

The discovery of MRT associated to SEC is rare: in a review, Babgi et al. describe a case of spinal MRT with an intra- and extramedullary lesion in the thoracolumbar region, treated with a combination of surgery, systemic chemotherapy and radiotherapy; the patient survived for about 16 months. An additional 25 cases with spinal structure involvement are reported in the review. Radiological characterization was available in only 11 cases, 10 of which were described as extramedullary intradural lesions and one as extramedullary intradural lesion. Management was reported for only 16 patients: surgery was performed in 11, adjuvant chemotherapy was administered to all, and nine received radiotherapy. Survival information was reported in 17 cases; 13 died, the median survival time being 10 months (22). In addition, a 2016 paper describes 100 cases of extracranial RT, 14 of which had a paraspinal localization and one had metastatic disease. This cohort included 13 patients with congenital RT, all of whom died. Five of these had metastatic disease and most had a lesion >5 cm. The main sites of primary disease were: paraspinal, thoracic, retroperitoneal, and hepatic. It is possible that these patients had a germline mutation of the *SMARCB1* gene, but these data are not present in the review (20).

The presence of dysmorphic features in a newborn diagnosed with a tumor prompted us to perform appropriate genetic analyses. To exclude RT predisposition syndrome (23), genomic DNA was isolated from the patient's peripheral blood and analysis of the coding sequenceof INI1gene was carried out. Constitutional DNA was used for MLPA analysis to detect abnormal copy numbers of the gene locus. Neither MLPA analysis nor HSNF5/INI1 gene sequencing on the patient's constitutional DNA revealed genetic alterations, thus excluding RT predisposition syndrome. Array CGH analysis on the child's DNA identified a 10q21.3 deletion of 81.28 kb, transmitted by her healthy mother. This deletion interval could have been responsible for the patient's dysmorphic features, and the same overlapping interval could account for both developmental delay and mental disability (24). This deletion interrupted the *CTNNA3* gene, which is the most recently characterized member of the alpha catenin family*. CTNNA3*, a cell-cell adhesion gene, encodes a protein of 895 amino acids; in hepatocellular carcinoma (HCC), this works as a tumor suppressor (25). *CTNNA3* is reported to inhibit the proliferation, migration and invasion of HCC cell lines. In addition, the mother's DNA carried a 3p13 deletion of 68.92 kb (71772963–71841921) containing the *PROK2* gene, a gene responsible for type-4 Kallman syndrome, or isolated GnRH deficiency without anosmia (26), although the mother is healthy and normosomic.

In conclusion, we describe the case of a newborn who, at birth, presented dysmorphic features and neurological deficit of limbs secondary to invasion of the spinal canal by a paravertebral tumor mass, which turned out to be a malignant rhabdoid tumor. As expected, despite prompt specific treatment, the clinical course was rapidly fatal.

## Data Availability

The original contributions presented in the study are included in the article/Supplementary Material, further inquiries can be directed to the corresponding author.
